# The Current Insecticide Resistance in Main Malaria Vector *Anopheles arabiensis* in Yemen

**DOI:** 10.1155/2020/5625019

**Published:** 2020-03-30

**Authors:** Zalalham Al-Koleeby, Ahmed El Aboudi, Mithaq Assada, Mohamed Al-Hadi, Mohammed Abdalr Ahman, Abdullah Awash, Abdul Samad Ahmed, Hani Mohamedi, Jamil Al Jarbany, Chafika Faraj

**Affiliations:** ^1^Plant and Microbial Biotechnology, Biodiversity and the Environment, Faculty of Science, Agdal University, Rabat, Morocco; ^2^Laboratory of Medical Entomology, National Institute of Hygiene, Rabat, Morocco; ^3^National Malaria Control Program, Ministry of Health and Population, Sana'a, Yemen

## Abstract

Control of malaria vectors in Yemen relies on both indoor residual spraying using carbamate (bendiocarb) and long-lasting pyrethroids-treated nets. This paper reports the results of studies conducted to monitor the insecticide resistance of the main malaria vector, *Anopheles arabiensis*, to the insecticides currently used in the vector control in four different locations. Susceptibility tests were performed following the WHO test procedures. Two pyrethroids (lambda-cyhalothrin 0.05% and deltamethrin 0.05%) and one carbamate (bendiocarb 0.1%) were tested at diagnostic doses (DD). The five-fold DD of lambda-cyhalothrin and deltamethrin (0.25%) were also used to yield information on the intensity of resistance. Besides, tests with synergists were performed to assess the involvement of detoxifying enzyme in the phenotypic resistance of the populations of *An. arabiensis* to pyrethroids. The results of the performed susceptibility bioassay showed that the vector is susceptible to bendiocarb and resistant to lambda-cyhalothrin and deltamethrin in the four studied areas. The pyrethroids resistance is solely metabolic. This information could help policy-makers to plan insecticide resistance management. Bendiocarb is still an effective insecticide in the form of IRS. Concerning LLINS, it would be interesting to assess their effectiveness, combining a pyrethroid with PBO for the control of the pyrethroid-resistant malaria vector.

## 1. Introduction

Malaria is a significant public health problem in Yemen with 65% of the population living in areas where transmission is thought to occur resulting in an estimated one million cases per year and a case fatality rate of about 1% [1]. The predominant parasite species is *Plasmodium falciparum*, and the overall prevalence of infections was 1.5% in 2009, with 97% of infections occurring in governorates predominantly below altitudes of 1000 m [2]. Transmission is maintained largely by *Anopheles arabiensis*, while *An. culicifacies* and *An. sergenti* are considered important vectors, respectively, in coastal and highland areas [3].

Control of malaria vectors in Yemen relies on indoor residual spraying (IRS) and use of long lasting insecticide-treated nets (LLINs). IRS was first launched in 2001 while distribution of LLINs began the following year. Two cycles of IRS are targeted in all areas below 600 m altitude (Stratum 1), one cycle of IRS supplemented with LLINs in areas from 600 m to 1000 m altitude (Stratum 2) and LLINs only in areas from 1000 to 1500 m altitude (Stratum 3). The residual insecticides used for IRS were lambda-cyhalothrin (ICON 10 wp and ICON 10 CS) until 2014 after which a shift was made to using bendiocarb (FICAM 80% WP). The LLINs distributed for populations have included the following product names and types: Netprotect® (deltamethrin), PermaNet® 2.0 (deltamethrin), Royal Sentry® (alpha-cypermethrin), and Yorkool® (deltamethrin) [4].

The first susceptibility tests were conducted in 2009. Results of these tests showed the full susceptibility of *An. arabiensis* to lambda-cyhalothrin in ten sites, to permethrin in two sites, to deltamethrin in one site, to bendiocarb in one site, and to DDT in four sites [5]. The first pyrethroid resistance was detected in 2012-2013 in several localities: in Al Qanawis (Stratum 1), resistance to lambda-cyhalothrin and permethrin and in Wadi Sulkmal (Stratum 2), and Zabied (Stratum 1), resistance to lambda-cyhalothrin and deltamethrin [5]. Since that, no further investigation has been conducted and resistance mechanisms have not been identified. However, the pyrethroid resistance detected represents a threat for malaria vector control strategy. The National Malaria Control Program (NMCP) needs data on the status of insecticide resistance in vector populations in different malaria ecoepidemiological areas, to be used as a guide to the choice of malaria vector control and resistance strategies. This study was developed in this context. It aims to evaluate and characterize the current insecticide resistance of the main malaria vector, *An. Arabiensis,* in Yemen, to the insecticides currently used in the vector control.

## 2. Materials and Methods

### 2.1. Study Area

**Figure 1:**
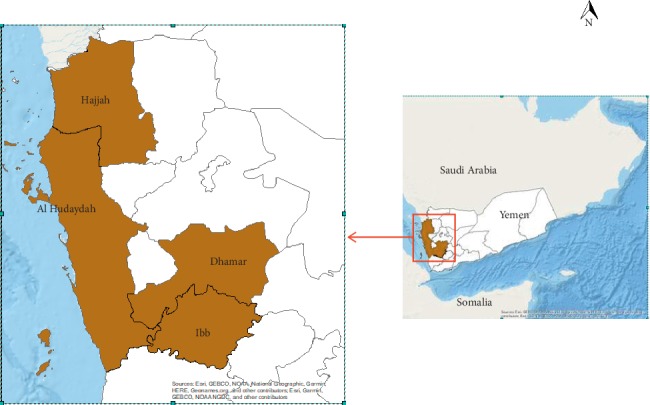
Map showing the location of the study sites.

The study was carried out in four villages, belonging to the most epidemic areas in Yemen, and where malaria vector control interventions are different. The selected sites are as follows (Figure 1):

1. Kabat Asharif (14,370018°–43,511675° and alt: 351 m) in Wusab As Safil province, Dhamar governorate. This site belongs to the epidemiological Stratum 1
2. Almahd Ala'asfal (14,824385°–43,225418° and alt: 120 m) in Bajil province, Hudaydah governorate (Stratum 1)
3. Arighah and Al Zaghabiah (15,590632°–43,28948° and alt: 340 m) in BaniQais province, Hajjah governorate (Stratum 1)
4. Asfal Hairan (13,905475°–43,770546° and alt: 902 m) in Fara'a Al Udayn province, Ibb governorate (Stratum 2)

The climate of Al Hudaydah is tropical desert, with only 80 millimeters of rainfalls every year. Despite the rare rains, this region includes unhealthy and malarious areas because of the rivers which descend from the mountains and feed certain marshes. Contrariwise, the climate in Dhamar, Ibb, and Hajjah is warm and moderately rainy. The city of Ibb receives up to 1000 mm of rain a year.

### 2.2. Mosquito Collection

*Anopheles arabiensis* larvae and pupae were collected based on a dipping method during the winter season, from October to December of 2017, from natural breeding sites of each district. Mosquito larvae were transported to the insectaria and maintained in rearing conditions until adult stages. The collected mosquitoes were identified morphologically [6], and only females of *An. arabiensis* were selected for bioassays.

### 2.3. Insecticide Bioassay

Susceptibility tests were performed, under optimum conditions (temperature 25–27°C and relative humidity 70%–80%), using the standard WHO bioassay method following the WHO test procedures [7]. The WHO kits (insecticide-impregnated papers) were supplied from University Sains Malaysia.

Three insecticides were tested at diagnostic doses (DD) as defined by the WHO [7]: two pyrethroids (lambda-cyhalothrin 0.05% and deltamethrin 0.05%) and one carbamate (bendiocarb 0.1%). The five-fold DD (5xDD) of lambda-cyhalothrin and deltamethrin (0.25%) were also used to yield information on the intensity of resistance. Four batches of 15–25 unfed females, aged 3 days, were exposed to impregnated papers for 1 h. The knockdown of the exposed mosquitoes to all insecticides was recorded after 1 hour of exposure. Batches of 50 mosquitoes exposed to untreated papers were used as control. Mortalities were recorded after 24 hours, and susceptibility status of mosquito populations was graded according to the WHO protocol [7].

### 2.4. Tests with Synergists

In order to assess the involvement of detoxifying enzyme in the phenotypic resistance of the populations of *An. arabiensis* to pyrethroids, tests with synergists were performed. Unfed females aged 2–4 days were preexposed to PBO 4% for 1 h before they were exposed to papers impregnated with lambda-cyhalothrin for 1 h. The control mosquitoes were preexposed with nonimpregnated papers for 1 h. Mosquitoes were transferred to observation tubes, supplied with sugar solution, and held for 24 h before recording mortality. Two replicates of 25–30 mosquitoes were exposed to each treatment.

### 2.5. Data Analysis

Percentage mortality was calculated from the results of the bioassay. The WHO criteria were adopted for distinguishing between the resistance/susceptibility status and the intensity of resistance of the tested mosquito populations [7]. When more than 98% mortality at DD was observed, the population was considered susceptible and when less than 90% mortality was observed, the population was considered “resistant.” Mortality of 98–100% at the 5xDD indicates low resistance intensity. Mortality of <98% indicates moderate resistance intensity.

In regard to the population pretreated by PBO 4%, when mean mortality was ≥98% in the “PBO followed by insecticide” samples, this implies that a monooxygenase-based resistance mechanism fully accounts for expression of the resistant phenotype in the test population. When mean mortality in the “PBO followed by insecticide” samples is superior than mean mortality in the “insecticide-only” samples but <98%, the phenotype resistance observed is partially due to a monooxygenase-based resistance mechanism and other resistance mechanisms are likely to be present in the test population. When mean mortality in the “PBO followed by insecticide” samples is ≤ mean mortality in the “insecticide-only” samples, the resistance detected is not based on monooxygenase-mediated detoxification.

## 3. Results

The results of insecticide susceptibility bioassay of the field population mosquitoes of *An. arabiensis* collected in the four study areas of Yemen in 2017 are indicated in Table 1.

With bendiocarb 0.1%, the mortality rates observed in all tested populations exceeded 98%, thus showing full susceptibility to this insecticide. However, the effect of lambda-cyhalothrin was lower. Mortality rates to DD (0.05%) ranged between 67% and 90%, which are below the susceptibility threshold of 98%. The assessment of the intensity of the observed resistance showed low resistance in all *An. arabiensis* populations from the four studied sites.

Resistance to deltamethrin in mosquito populations from Kabat Asharif (Dhamar), Asfal Hairan (Ibb), and Almahd Ala'asfal (Al Hudaydah) was observed with respective mortalities of 88, 90, and 93%. The assessment of intensity of this resistance in Almahd Ala'asfal population showed a moderate resistance with 96% of mortality to 5xDD. The pyrethroid-resistant population of *An. arabiensis* from Almahd Ala'asfal was tested by the synergist PBO 4% to assess the potential role played by Cytochrome P450 monooxygenases in resistance observed. After preexposure to PBO, the susceptibility to lambda-cyhalothrin 0.05% was fully restored. The mortality obtained with delthamethrin alone was 74%, after exposure to PBO, and this mortality increased to 100%.

## 4. Discussion

The objective of this study was to characterize and quantify the intensity of the major malaria vector resistance to the insecticides currently used in vector control strategies in Yemen. It showed that *An. arabiensis* is fully susceptible to bendiocarb but resistant to pyrethroids in the four mosquito populations tested. The intensity of resistance was low to lambda-cyhalothrin in the four sites investigated and moderate to deltamethrin in Almahd Ala'asfal.

Our results about the state of susceptibility to carbamates are consistent with the local data of International Organization for Migration that reported in 2015 that vectors are susceptible to carbamates. This is the insecticide used for IRS in 2016-17 in Yemen [8]. In the other hand, the evidence for resistance in *An. arabiensis* to pyrethroids is of concern, deducing that vector control using these insecticides in Yemen may be compromised and highlighting a particular need to develop an appropriate resistance management strategy.

The preexposure to PBO restored full susceptibility to lambda-cyhalothrin in the Almahd Ala'asfal site, suggesting the involvement of a metabolic resistance as the sole mechanism of pyrethroid resistance detected. PBO affects monooxygenase activity; therefore, results of this bioassay indicate the involvement of monooxygenases in resistance [9].

Resistance to pyrethroids in Yemen may be mainly due to the widespread use of these insecticides for public health programs since 2001 in IRS and LLINs mass distribution campaigns [4]. Lambda-cyhalothrin was used for IRS in two cycles per year from 2001 to 2013 in the Wusab Assafil, Arighah, and Almahd Ala'asfal regions and, in one cycle, in the Asfal Hairan region. In 2014, it was replaced by bendiocarb. Delthamethrin has always been used with alpha-cypermethrin, prior to the onset of political instability, for LLINs in the Asfal Hairan region [8]. The wide use of pyrethroids in the four investigated areas had led to a selective pressure on *An. arabiensis* and might explain the similar resistance intensity between the four regions.

This resistance may be also due to the use of these insecticides within agricultural practices that represent the main activities of many districts. In fact, the northern mountainous areas of Yemen, including Sanâa, are where most of the imported pesticides are used.

In order to act before insecticide resistance compromises current vector control strategies, the WHO has proposed various guidelines to encourage countries to plan and implement insecticide resistance management strategies [9]. Operationally, when the resistance is detected for an insecticide, the main gateway for vector control programs is the use of other insecticide categories that may reduce selection pressure and spread of resistance. Four classes of insecticides, namely, pyrethroids, organochlorine (solely DDT), organophosphates, and carbamates are used in the public health control program. However, pyrethroids and DDT share similar modes of action, thus making organophosphate and carbamate very important in resistance management strategies. Various resistance management strategies including rotations, mosaics, and mixture of pyrethroid and/or organophosphate or carbamate for IRS and on nets have been demonstrated [10–12]. Therefore, knowledge on involved resistance mechanism is important.

This study shows that the *An. arabiensis* resistance to lambda-cyhalothrin is only metabolic and maybe due to overactivity of monooxygenases. Three families of metabolic enzymes are associated with resistance in malaria vectors: monooxygenases (P450s), esterases, and glutathione-S-transferases (GSTs) [9]. Edi et al. [13] demonstrated that amplified expression of multiple monooxygenases was related to pyrethroid resistance. Esterase-mediated resistance has been presented to reduce susceptibility of malaria vectors to both organophosphates and pyrethroids [14], and increased expression of GSTs has been associated with DDT resistance [15]. These enzyme systems may also have a broad spectrum of activity and be capable of detoxifying a range of insecticides. For instance, monooxygenases can detoxify pyrethroids and also carbamates [9]. This is of concern, as it could affect the future of bendiocarb residual spraying as an alternative to lambda-cyhalothrin in the control of malaria vector in Yemen.

Unfortunately, LLINs are highly dependent on a single class of insecticides, the pyrethroids. They are the only insecticide group recommended by the World Health Organization for treatment of mosquito nets due to their rapid knockdown effect and relatively lower mammalian toxicity [9, 16]. The PBO synergist, acts by increasing the impact of pyrethroids by inhibiting metabolic enzymes in the mosquitoes, making them susceptible to the pyrethroid [9]. Several studies have shown the effectiveness of LLINs impregnated simultaneously with synergists on malaria transmission [17] and on resistance management. Due to great concerns brought about by the possible failure of control interventions using LLINs in Yemen, PBO LLINs can be suggested as a resistance management strategy.

## 5. Conclusion

*Anopheles arabiensis* is susceptible to bendiocarb and resistant to lambda-cyhalothrin and deltamethrin in the four studied areas in Yemen. This pyrethroid resistance is solely metabolic.

This useful information could help policy-makers to plan insecticide resistance management. Bendiocarb is still an effective insecticide in the form of IRS. Concerning LLINs, it would be interesting to assess the effectiveness of LLINs, combing a pyrethroid with PBO for the control of pyrethroid-resistant malaria vectors in Yemen.

## Figures and Tables

**Table 1:** Bioassay mortality of the field population of *An. arabiensis* to lambda-cyhalothrin 0.05% and 0.25%, deltamethrin 0.05% and 0.25%, and bendiocarb 0.1%.

		Regions															
		Kabat Asharif				Arighah				Almahd Ala'asfal				Asfal Hairan			
Insecticide	Dose (%)	*N*	% M	% Kd	St	*N*	% M	% Kd	St	*N*	% M	% Kd	St	*N*	% M	% Kd	St

Bendiocarb	0.1	102	99	99	S	60	100	100	S	86	100	100	S	171	99		S
Lambda-cyalothrin	0.05	114	88	100	R	78	90	92	R	73	82	93	R	181	67	80	R
Lambda-cyalothrin	0.25	147	100	74	LR	81	100	97.5	LR	129	98	100	LR	107	100	100	LR
Deltamethrin	0.05	104	88	100	R	—	—	—	—	97	93	96	R	98	90	91	R
Deltamethrin	0.25	—	—	—	—	—	—	—	—	112	96	100	MR	—	—	—	—

*N,* total number of mosquitoes; %M, percentage of mortality; %Kd, knockdown percentage; St, resistance status.

## Data Availability

The data used to support the findings of this study are available from the corresponding author upon request.
